# Tolerance and overcompensation to infection by *Phytophthora infestans *in the wild perennial climber *Solanum dulcamara*

**DOI:** 10.1002/ece3.5057

**Published:** 2019-04-05

**Authors:** Laura Masini, Laura J. Grenville‐Briggs, Erik Andreasson, Lars Råberg, Åsa Lankinen

**Affiliations:** ^1^ Plant Protection Biology Swedish University of Agricultural Sciences Alnarp Sweden; ^2^ Department of Biology Lund University Lund Sweden; ^3^ Present address: British American Tobacco Plant Biotechnology Division Cambridge UK

**Keywords:** host‐pathogen interactions, late blight, overcompensation, *Phytophthora infestans*, *Solanum dulcamara*, tolerance

## Abstract

Studies of infection by *Phytophthora infestans*—the causal agent of potato late blight—in wild species can provide novel insights into plant defense responses, and indicate how wild plants might be influenced by recurrent epidemics in agricultural fields. In the present study, our aim was to investigate if different clones of *Solanum dulcamara* (a relative of potato) collected in the wild differ in resistance and tolerance to infection by a common European isolate of *P. infestans*. We performed infection experiments with six *S. dulcamara *genotypes (clones) both in the laboratory and in the field and measured the degree of infection and plant performance traits. In the laboratory, the six evaluated genotypes varied from resistant to susceptible, as measured by degree of infection 20 days post infection. Two of the four genotypes susceptible to infection showed a quadratic (concave downward) relationship between the degree of infection and shoot length, with maximum shoot length at intermediate values of infection. This result suggests overcompensation, that is, an increase in growth in infected individuals. The number of leaves decreased with increasing degree of infection, but at different rates in the four susceptible genotypes, indicating genetic variation for tolerance. In the field, the inoculated genotypes did not show any disease symptoms, but plant biomass at the end of the growing season was higher for inoculated plants than for controls, in‐line with the overcompensation detected in the laboratory. We conclude that in *S. dulcamara* there are indications of genetic variation for both resistance and tolerance to *P. infestans *infection. Moreover, some genotypes displayed overcompensation. Learning about plant tolerance and overcompensation to infection by pathogens can help broaden our understanding of plant defense in natural populations and help develop more sustainable plant protection strategies for economically important crop diseases.

## 1 INTRODUCTION

*Phytophthora infestans* is an oomycete that causes potato late blight, which is the most detrimental disease of cultivated potato worldwide. Late blight has been a serious disease in Europe since the Irish potato famine in the 1840s. Today it results in annual economic losses worth at least 5 billion US dollars globally and a strong dependence on synthetic or copper‐based fungicides (Haverkort, Struik, Visser, & Jacobsen, 2009). Developing host plant resistance is believed to be the most effective and environmentally sound method for controlling the disease. However, since *P. infestans* is highly adaptable and able to overcome host resistance (Haas et al., 2009), it is essential to find alternative ways to combat this pathogen, which should ideally be combined into an integrated disease management strategy. Studying wild crop relatives can be important in this context both in relation to identifying novel defense strategies (Rodewald & Trognitz, 2013) and to better understand how these plants could influence epidemiology of the disease (Stam & McDonald, 2018; Thrall et al., 2011). Moreover, from an ecological and evolutionary biology perspective it is also of interest to understand how wild plants interact with crop diseases that cause annual epidemics in agricultural fields (Burdon & Thrall, 2014; Upson, Zess, Białas, Wu, & Kamoun, 2018).

Resistance and tolerance represent two different aspects of host defense against pathogens, where resistance is the ability to limit pathogen proliferation during infection, while tolerance is the ability of the plant to restrict the harm or fitness reduction caused by a given pathogen load (Råberg, Graham, & Read, 2009; Rausher, 2001; Stowe, Marquis, Hochwender, & Simms, 2000). Unlike resistance, tolerance to pathogens, has not been well studied, particularly in wild crop relatives that are also able to harbor crop diseases (but see Caldwell, Schafer, Compton, & Petterson, 1958; Gurney, Press, & Scholes, 2002; Koch, Chapman, Louis, Heng‐Moss, & Sarath, 2016). It is important to distinguish between resistance and tolerance, as they are expected to have profoundly different influences on pathogen‐host coevolution (Best, White, & Boots, 2014; Bingham, Walters, Foulkes, & Paveley, 2009; Rausher, 2001). Because resistance protects the host by limiting pathogen replication, this type of defense will select for counter‐adaptations in/of the pathogen to help it overcome the host defenses. Tolerance, on the other hand, should not have direct negative effects on the pathogen, and therefore, not impose selection for counter‐adaptations (Rausher, 2001; but see Vale, Fenton, & Brown, 2014). Identifying tolerance mechanisms could, therefore, have important implications, for the development of sustainable pest management tools for crops attacked by severe and rapidly evolving pests or pathogens (Mitchell, Brennan, Graham, & Karley, 2016; Peterson, Varella, & Higley, 2017).

It is well established that genetic variation exists not only for resistance but also for tolerance (Fineblum & Rauscher, 1995; Koskela, Puustinen, Salonen, & Mutikainen, 2002; Roux, Gao, & Bergelson, 2010; Simms & Triplett, 1994; Stowe et al., 2000). In particular tolerance to herbivory has been studied extensively (Koch et al., 2016; Mitchell et al., 2016; Núñez‐Farfán, Fornoni, & Valverde, 2007; Strauss & Agrawal, 1999). The study of tolerance to pests and pathogens is complicated by the difficulty of defining and quantifying tolerance (Peterson et al., 2017; Stowe et al., 2000). For example, while tolerance to infection by pathogens has been identified in a number of study species and crops (Kover & Schaal, 2002; Parker, Welham, Paveley, Foulkes, & Scott, 2004; Politowski & Browning, 1978; Simms & Triplett, 1994; see also review by Pagán & García‐Arenal, 2018), the definitions used for tolerance to infection are often inconsistent (Bingham & Newton, 2009; Castro & Simón, 2016).

To accurately quantify tolerance, it is proposed that the reaction norm between fitness or yield and the degree of infection should be evaluated (Fineblum & Rauscher, 1995; Råberg et al., 2009; Simms, 2001; Stowe et al., 2000). A reaction norm is the pattern of phenotypic expression of one genotype across a range of environments (Schlichting & Pigliucci, 1998), which means that tolerance cannot be measured in one individual plant but rather must be assessed in several replicates of the same genotype that differ in their degree of infection. Besides eliminating confounding factors like “general vigor,” this way of measuring tolerance also allows for more detailed evaluation of the trait, such as nonlinear relationships between fitness and infection percentage (Råberg et al., 2009; Simms, 2001). This approach can also facilitate detection of “overcompensation,” which is a special case of tolerance where plant interactions with pests and pathogens lead to an increase in fitness (Agrawal, 2000).

In this study, our overall aim was to test if different clones of *Solanum dulcamara*, a wild relative of cultivated potato (*Solanum tuberosum)*, differ in response to infection by *P. infestans* in a whole‐plant infection system. *Solanum dulcamara* is an herbaceous, perennial vine that grows in a wide range of habitats, from woodland to scrubland, hedges and wetlands, and is often found in close proximity to agricultural land. Natural infection by *P. infestans* has been reported mainly in *S. dulcamara* growing close to potato fields (Cooke, Carlisle, Wilson, & Deahl, 2002; Flier, van den Bosch, & Turkensteen, 2003). Infection was also seen in inoculation experiments using *P. infestans* isolates originally collected from *S. dulcamara* as well as from potato and other wild and introduced hosts (*Solanum nigrum* and *Solanum sisymbriifolium *(Flier et al., 2003). Previous studies have shown that *S. dulcamara* genotypes vary from resistant to susceptible to *P. infestans* (Abreha, Lankinen, Masini, Hydbom, & Andreasson, 2018; Golas et al., 2010). This variation was consistent for three isolates with a broad spectrum of virulence collected from the Netherlands and Belgium (Golas et al., 2010). In a recent study of 12 populations from the south of Sweden in an area with intensive potato farming (Eriksson, Carlson‐Nilsson, Ortíz, & Andreasson, 2016), we identified four resistance types using detached leaf assays: (a) resistant without any disease symptoms; R, (b) resistant with small necrotic lesion, R^N^; (c) susceptible with expanding lesions, S^L^; and (d) susceptible with visible sporulation, S (Abreha et al., 2018). Here, we performed whole‐plant infection experiments in the laboratory and in the field, using clones of six plant genotypes. We estimated the relationship between plant performance traits representing vegetative growth and degree of infection as an indication of tolerance using a low and a high level of inoculum to produce large variation in infection among clones. A significant interaction between degree of infection and plant genotype influencing plant performance would indicate genetic variation for tolerance (Råberg et al., 2009; Simms, 2001).

We asked: (a) Do *S. dulcamara* clones differ in tolerance to infection? (b) Do field‐grown plants show the same relationship between infection and plant performance as plants grown in the laboratory? (c) Are the early measured performance traits estimated in the laboratory indicative of later performance in the field?

## 2 MATERIALS AND METHODS

### 2.1 Plant material

**Figure 1:**
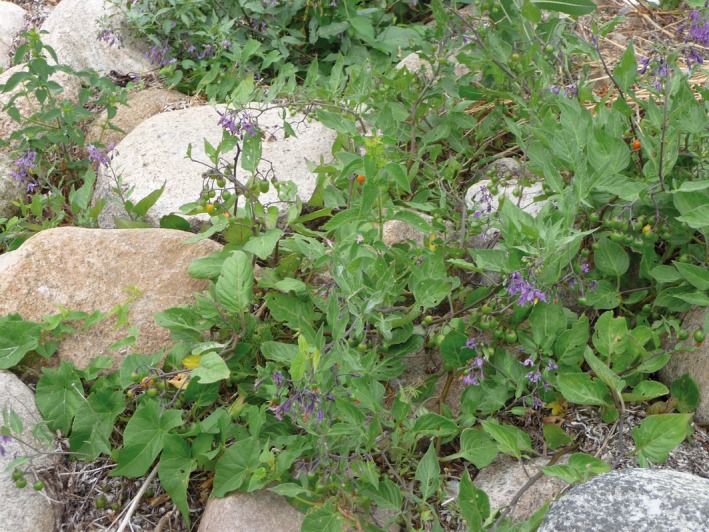
*Solanum dulcamara* in a natural population. Photograph: Åsa Lankinen

*Solanum dulcamara *L (Solanaceae), bittersweet, is native to Europe and Asia, and commonly found in Swedish wetlands, ruderal land and urban areas (Mossberg & Stenberg, 2003). Plants grow shoots that can be up to 4 m long, but usually reach 1–2 m (Figure 1). The purple flowers with yellow anthers are produced in loose clusters. Flowers develop into red berries.

For the purpose of the current study, we selected six genotypes from four of the previously screened 12 populations: Geneticum (G) in an urban hedgerow adjacent to a parking lot in the campus of Lund University in the city of Lund; Lomma2 (L2) in a wooded area along a road close to the sea outside the town Lomma; Lomma3 (L3) on the beach outside the town Lomma: Dalby (D) in a forest outside of the village Dalby (Abreha et al., 2018). These genotypes differed in resistance phenotypes determined by detached leaf assays, involving resistant with small necrotic lesion (R^N^), susceptible with expanding necrotic lesion (S^L^), and susceptible with visible sporulation (S). While quantitative PCR confirmed the presence of *P. infestans* in all three resistance phenotypes, providing a potential system for the study of tolerance, the abundance of *P. infestans* differed among phenotypes (Abreha et al., 2018).

We propagated plants in vitro by stem cuttings and grew them for 4 weeks in a shooting‐medium (MS, Murashige & Skoog, 1962), modified according to (Abreha, Alexandersson, Vossen, Anderson, & Andreasson, 2015). We transplanted clones to soil (standard compost, 1.5 L pots) and covered with a plastic cup for 1 week. After 4 weeks in a growth chamber (20°C, 16:8 hr light:dark cycle and 70% relative humidity), plants were used for infection experiments.

### 2.2 Preparation of inoculum for infection experiments

The *P. infestans *isolate 88069 (A1 mating type race 1.3.4.7) was used throughout this study. This isolate was originally obtained from tomato in the Netherlands (Pieterse, Risseeuw, & Davidse, 1991) and is considered the standard European reference strain. This well‐studied isolate was used for three reasons: (a) due to the ease in which consistent sporangial production is achieved in the laboratory with isolate 88069; (b) due to the large body of molecular and genetic data that exists from this isolate; and (c) since field tests were to be performed and highly aggressive European isolates from the Pink6 and Blue13 clonal lineages (Cooke et al., 2012) are absent from Sweden (Yuen & Andersson, 2013), we were not able to obtain permission for field work with these isolates. However, preliminary data obtained in the laboratory showed similar rates of infection when an isolate of the Pink6 clonal lineage (6_A1) was tested or when a local Swedish isolate was used (data not shown). Our aim was also to choose an isolate with medium levels of aggressiveness so that we could increase the range of degree of infection (pathogen load), and thereby increase the power to detect variation in tolerance to infection (Råberg et al., 2009).

*Phytophthora infestans *was grown on rye‐pea or V8 agar as previously described (Vetukuri, Whisson, & Grenville‐Briggs, 2017). It was passaged through susceptible potato leaf discs twice prior to use in all experiments to ensure pathogenicity was maintained. Sporangia were harvested in sterile tap water, adjusted to a concentration of 15,000 (low) or 30,000 (high) sporangia/ml, using a hemocytometer, and incubated at 4°C for 2 hr to promote zoospore release prior to infection. Similar to selecting an isolate with medium aggressiveness, different inoculum concentrations were used to increase the range of degree of infection (pathogen load). The high inoculum treatment was chosen in order to achieve 100% infection. Thus, we used the standard (EuroBlight protocol) number of sporangia routinely used in the lab as our lower value and a twofold increase to represent our higher infection level.

### 2.3 Controlled infection experiments in the laboratory

To investigate genetic variation for tolerance to infection with *P. infestans*, we conducted 13 separate experiments under controlled conditions in the laboratory involving six *S. dulcamara *genotypes (Table 1). For logistical reasons, the experiments were performed in two batches, with three genotypes in each batch (Table 1). While we used both control plants (*N* = 3–5) and inoculated plants (*N* = 8–10) in each experiment, the number of clones varied depending on the availability of propagated in vitro plants (Table 1).

**Table 1:** List of experiments performed in the laboratory under controlled conditions and in the field to investigate genetic variation in tolerance to *Phytophthora infestans* in six genotypes of *Solanum dulcamara*

Batch	Experiment month, year	Inoculum	Number of plants sprayed (control) per genotype					
			G:20.1	G:21.1	L2:3.6	D:5.1	L2: 3.2	L3: 4.1
Laboratory								
1	Aug 2015	H	5 (2)	5 (3)	5 (1)			
1	Dec 2015	L		11 (3)	12 (3)			
1	March 2016	L	10 (3)	12 (3)	11 (3)			
1	April 2016	L		5 (5)	7 (5)			
1	May 2016	H	8 (5)	10 (5)	10 (5)			
1	Feb 2017	L, H		3 (4)	9 (5)			
1	March 2017	L, H	7 (5)	10 (5)	9 (5)			
2	Aug 2016	L, H				10 (5)	10 (5)	10 (5)
2	Sept 2016	L, H				8 (5)	10 (5)	7 (4)
2	Oct 2016:1	L, H				8 (5)	9 (5)	10 (5)
2	Oct 2016:2	L, H					10 (4)	10 (5)
2	Nov 2016	H					5 (5)	5 (5)
2	Dec 2016	L, H				8 (5)	10 (5)	10 (4)
Field								
1	July‐Sept 2016	H	10 (7)	20 (10)	20 (10)			

Note

L = 15,000 sporangia per ml, H = 30,000 sporangia per ml.

Infection experiments were carried out as previously described (Ali et al., 2012). One day before inoculation, plants were placed in an infection growth room with 100% relative humidity. Plants were sprayed with either high or low inoculum (30,000 sporangia/ml or 15,000 sporangia/ml respectively) or with sterile water as a control (Table 1). Relative humidity of 100% was maintained for 2 days post inoculation (dpi) and thereafter adjusted to 90% for the remainder of the experiment.

At 20 dpi, we determined the degree of infection per plant using a standard screening method for late blight disease scoring in cultivated potato (Liljeroth, Bengtsson, Wiik, & Andreasson, 2010). We tested if this disease‐scoring key from potato would give representative scoring figures by confirming the quantification of infection using qPCR (Abreha et al., 2018). Plants were all checked for sporulation, which was included in the scoring. To measure plant performance traits, we calculated the length of all shoots, and the total number of open leaves on all shoots, per plant. To accurately evaluate plant growth during the experiment, the same measurements were taken both at 0 and 20 dpi. Performance traits were calculated by subtracting the 0 dpi values from those recorded at 20 dpi. Fresh weight in a subset of plants at 20 dpi correlated with both shoot length (both log‐transformed, Pearson *r = *0.456, *df* = 34, *p* = 0.005) and number of leaves (*r = *0.384, *df* = 34, *p* = 0.021).

### 2.4 Field trials

To test if the results of the controlled laboratory experiments correlated with field performance, we conducted a field trial involving three of the genotypes studied in the laboratory (G:20.1, G:21.1 and L2: 3.6; Table 1). The field study also allowed us to investigate the relationship between performance traits displayed earlier in the life‐cycle, that is, growth prior to reproduction, and those displayed later in the life‐cycle during reproduction and maturation, since it was not possible to observe traits occurring later in the plant life‐cycle in the controlled growth room experiments. We planted 17–30 in vitro clones per genotype distributed in six plots (2.5 × 2.5 m) in a randomised block design. Plants had grown in soil in the greenhouse for 4 weeks. We covered each plot with an insect net to prevent herbivory by insects. After 3 weeks, we sprayed plants (*N* = 10–20 per genotype) with *P. infestans* inoculum (30,000 sporangia/ml). We sprayed control plants (*N* = 7–10 per genotype) with water. Spraying was conducted in the evening between 18:00 and 19:00 (Central European Summer Time, CEST). Directly following spraying, we covered each plant with a plastic bag and sealed it to increase the local relative humidity in order to promote infection. Plants were aerated after 24 hr and at 2 dpi bags were removed. We repeated the spray inoculations at 7 dpi using the same method as described above. The reason for the repeated spraying was that no infection resulted after the first spraying.

As for the laboratory experiment under controlled conditions, we measured the length of all shoots and counted all fully open leaves at 0 and 20 dpi. During the growing season, we counted the flowers produced, making sure to mark already counted flowers with thread to avoid double counting. The insect net was removed as plants started to flower to allow pollinator visits. At the end of the growing season at senescence, we harvested all the branches and estimated the dry weight of all shoots of each plant.

### 2.5 Statistical analysis

We performed statistical analyses in R (R Development Core Team, 2018), fitting general mixed‐effect models (type III sum of squares) in the packages lme4 (Bates, Maechler, Bolker, & Walker, 2015), lmertest (Kuznetsova, Brockhoff, & Christensen, 2017) and lsmeans (Lenth, 2016). When data were not normally distributed, we selected a suitable transformation method to obtain a normal distribution. All models were validated according to standard methods.

To test for genetic variation for resistance in the laboratory experiment, we used a model with degree of infection (plant infection percentage; arcsine‐square‐root transformed), against the fixed factors experimental treatment (control, or sprayed with two levels of inoculum), genotype and their interaction, and the random factor experiment; a significant effect of host genotype would indicate genetic variation for resistance. To test for genetic variation for tolerance, we used models with performance traits—total shoot length (log‐transformed) or total number of leaves of all shoots (20 − 0 dpi)—against the factor plant genotype, the continuous covariates infection percentage and the squared infection percentage (proportions were arcsine‐square‐root transformed), and their interactions with plant genotype (all fixed). A significant interaction between plant genotype and any of the continuous covariates would indicate genetic variation for tolerance. The continuous covariates were rescaled to a mean of zero and a standard deviation of 1. The random factor experiment was also included in the model. Each batch was analyzed separately.

Because field plants did not show any signs of infection after spraying, we tested differences in performance between sprayed and unsprayed plants rather than differences in tolerance. We included this test to investigate whether our laboratory data indicating overcompensation (see Results) was supported in the field. From the field, shoot length and number of leaves at 20 − 0 dpi, flower production and plant weight at harvest, were evaluated using a model with the fixed factors plant genotype, experimental treatment (control or sprayed), and their interaction. The random factor experimental block was also included. Eleven out of 76 plants lost shoots or shoots were broken at 20 dpi. These shoots were excluded from the measurements of the given plant.

## 3 RESULTS

### 3.1 Infection of *P. infestans* in multiple *S. dulcamara* genotypes under controlled conditions

**Figure 2:**
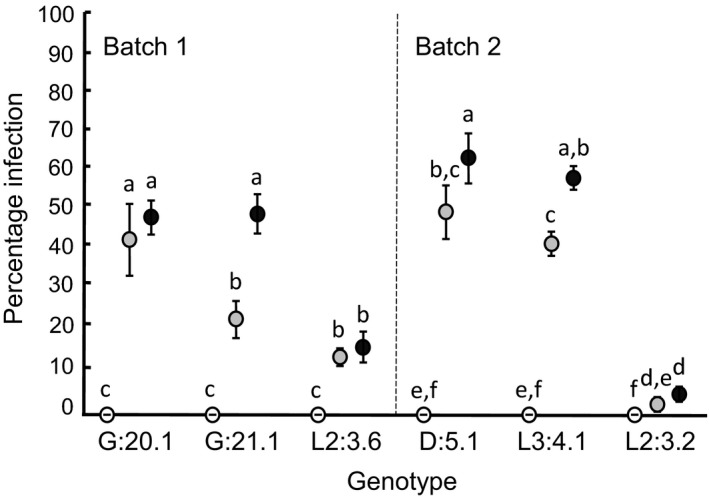
Degree of infection (percentage infection at 20 dpi) in six *Solanum dulcamara* genotypes treated with *Phytophthora infestans *inoculum of low (gray circles) or high (black circles) sporangia concentration in the laboratory under controlled conditions. Control plants = open circles. Infection experiments were conducted in two different batches and repeated 2–6 times per genotype and spore concentration (see Table 1). Error bars indicate standard error. Different letters indicate significant difference (*p* < 0.05) within batches

Plants sprayed with *P. infestans* inoculum of isolate 88069 in the laboratory had developed disease symptoms both for lower (15,000 per ml) and slightly higher (30,000 per ml) sporangia concentrations at 20 dpi (Figure 2). A higher spore concentration generally resulted in a higher degree of infection, but degree of infection also differed between genotypes as indicated by the significant genotype by treatment interaction (Table 2). Two of the genotypes (L2: 3.6 and L2: 3.2) showed relatively low degree of infection (<20%) for the high spore concentration (Figure 2).

**Table 2:** Analyses testing for variation in resistance among genotypes

Source of variation	Batch 1	Batch 2				
	*df*	*F*	*p*	*df*	*F*	*p*
Genotype	2,206	44.0	<0.001	2,205	287	<0.001
Treatment	2,209	240	<0.001	2,204	503	<0.001
Genotype × treat	4,205	17.8	<0.001	4,203	70.5	<0.001

Notes

General linear mixed models of degree of infection (percentage infection at 20 dpi calculated as arcsine‐transformed proportion) in control plants and plants treated with *Phytophthora infestans* at either low or high sporangia concentration. Six genotypes of *Solanum dulcamara *were tested in two batches in the laboratory under controlled conditions. Experiment = random factor included in the model.

### 3.2 Relationships between plant performance and *P. infestans* infection under controlled conditions

The two performance traits—total length and total number of leaves of all shoots (20 – 0 dpi)—showed a low, but significant positive correlation across all six genotypes (Pearson *r = *0.352, *df* = 442, *p* < 0.001).

**Figure 3:**
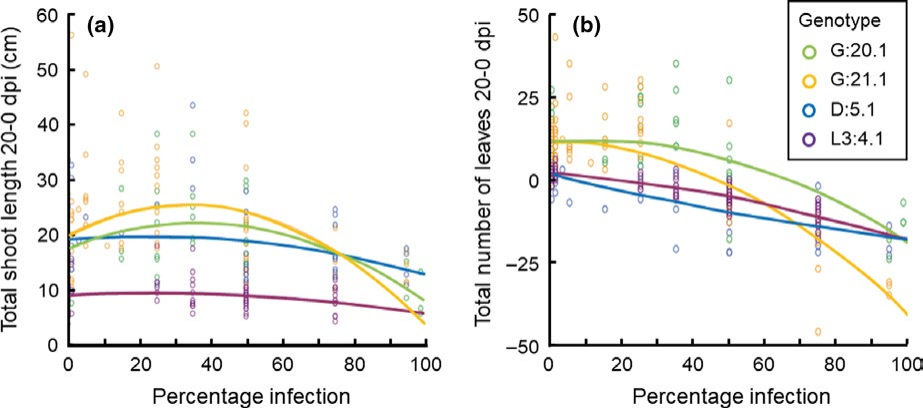
Plant performance in relation to degree of infection (percentage infection at 20 dpi) in control plants and plants treated with *Phytophthora infestans* in four susceptible genotypes of *Solanum dulcamara *grown in the laboratory under controlled conditions for 20 days. (a) Total shoot length of all shoots could be described by both a linear (not shown) and a quadratic relationship with percentage infection. (b) Total number of leaves of all shoots could be described by a quadratic relationship, which differed among genotypes. Performance traits were estimated at 20 dpi and corrected for the same measures at 0 dpi. Infection experiments were conducted in two different batches; batch 1: G:20.1 and G:21.1, batch 2: D:5.1 and L3:4.1

In the following analyses of the relationship between plant performance and degree of infection, we excluded the two highly resistant clones L2: 3.6 and L2: 3.2 since they showed low variation in infection (Figure 2). The four remaining clones with relatively low resistance (two clones per batch) varied extensively in infection severity at 20 dpi. In batch 1, there was both a linear and quadratic effect of degree of infection on total shoot length (Table 3, Figure 3a). At lower values of degree of infection, there was a positive relationship between shoot length and degree of infection, suggesting overcompensation (Figure 3a). At higher degrees of infection, shoot length decreased. In batch 2, only an effect of genotype was significant (Table 3). Interactions between genotype and degree of infection or the squared degree of infection were not significant for either batch (Table 3). This indicates that tolerance did not significantly differ between the investigated genotypes.

**Table 3:** Analyses testing for variation in tolerance among genotypes

Source of variation	Batch 1 (G 20.1, G 21.1)			Batch 2 (D 5:1, L3: 4.1)		
	*df*	*F*	*p*	*df*	*F*	*p*
Total shoot length 20–0 dpi (cm)						
Genotype	1,120	1.98	0.16	**1,127**	**155**	**<0.001**
Percentage infection	**1,120**	**13.2**	**<0.001**	1,125	1.57	0.21
Percentage infection^2^	**1,119**	**13.3**	**<0.001**	1,125	2.54	0.11
Genotype × Percentage infection	1,115	0.083	0.77	1,123	2.37	0.13
Genotype × Percentage infection^2^	1,114	0.097	0.76	1,123	2.79	0.097
Total number of leaves 20–0 dpi						
Genotype	**1,117**	**5.54**	**0.020**	**1,115**	**6.91**	**0.010**
Percentage infection	1,118	1.44	0.23	1,124	3.58	0.061
Percentage infection^2^	**1,119**	**4.21**	**0.042**	**1,118**	**6.37**	**0.013**
Genotype × Percentage infection	1,115	1.65	0.20	**1,126**	**6.45**	**0.012**
Genotype × Percentage infection^2^	**1,115**	**6.10**	**0.015**	**1,126**	**4.71**	**0.032**

Notes

Test of fixed effects in general linear mixed models of plant performance in relation to degree of infection (percentage infection at 20 dpi) in control plants and plants sprayed with *Phytophthora infestans* in four susceptible genotypes (two per batch) of *Solanum dulcamara *grown in the laboratory under controlled conditions. Experiment = random factor included in the model. Significant factors are given in bold. Total shoot length of all shoots (log‐transformed) and total number of leaves of all shoots was estimated 20 dpi and corrected for the same measures at 0 dpi.

The total number of leaves per plant was influenced by the squared degree of infection, but also by the interaction between plant genotype and the squared degree of infection in both batches (Table 3, Figure 3b). Thus, for this performance trait, our results suggest genetic variation in tolerance among the plant genotypes. In contrast to the result for shoot length, the number of leaves could not be described by a linear reduction of the degree of infection (Table 3), but in batch 2 the interaction between degree of infection and plant genotype was significant. Moreover, the number of leaves did not appear to increase for lower levels of infection (Figure 3b). The high loss of leaves at high levels of infection resulted in plants with fewer leaves (negative values) at the end of the experiment compared to initial values.

### 3.3 Field trials

**Figure 4:**
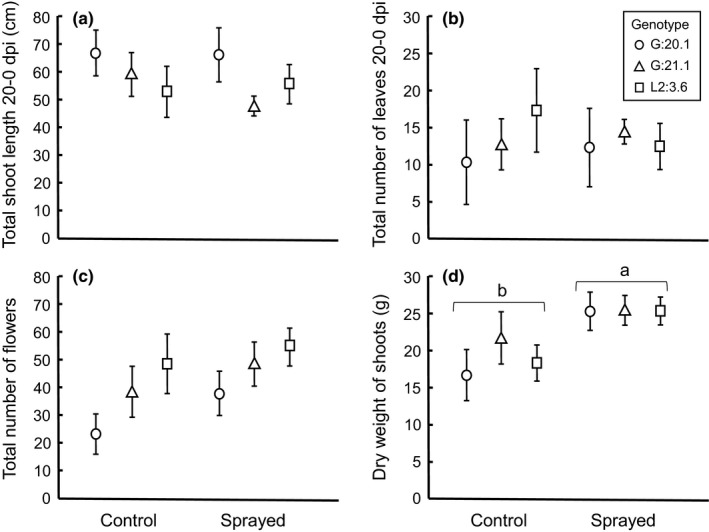
Four plant performance traits in control plants and plants treated with *Phytophthora infestans* in three genotypes of *Solanum dulcamara *grown in an experimental garden. (a) Total shoot length of all shoots and (b) total number of leaves of all shoots were estimated at 20 dpi and corrected for the same measures at 0 dpi. (c) Total number of flowers of all shoots over the season based on repeated estimates until early autumn. (d) Dry weight of all shoots estimated at harvest in the autumn when all leaves had fallen off. Error bars indicate standard error. Different letters indicate significant difference (*p* < 0.05)

There was no difference in total shoot length, total number of leaves of all shoots, or total number of flowers (20 – 0 dpi) between control and sprayed plants (Figure 4a–c; Table 4). However, the dry weight of shoots harvested in the autumn was significantly higher in sprayed plants than in controls of all genotypes (Figure 4d, Table 4). Despite spraying plants twice with inoculum with a high concentration of sporangia (30,000 per ml), we were unable to detect any disease symptoms during our repeated inspection of the plants.

**Table 4:** Analyses testing for variation in performance among genotypes in the field

Source of variation	Total shoot length 20–0 dpi (cm)			Total number of leaves 20–0 dpi			Total number of flowers			Dry weight of shoots (g)		
	*df*	*F*	*p*	*df*	*F*	*p*	*df*	*F*	*p*	*df*	*F*	*p*
Genotype	2,64	1.41	0.25	2,61	0.673	0.51	**2,67**	**22.4**	**<0.001**	2,66	0.553	0.58
Treatment	1,64	0.372	0.54	1,60	1.06	0.31	1,66	0.991	0.32	**1,66**	**8.00**	**0.006**
Genotype × treat	2,63	0.064	0.94	2,58	2.39	0.10	2,65	0.303	0.74	2,65	0.437	0.65

Notes

General linear mixed models of four plant performance traits in control plants and plants sprayed with *Phytophthora infestans* in three genotypes of *Solanum dulcamara *grown in an experimental garden in a block design. Block = random factor included in the model. Significant factors are given in bold. Total shoot length of all shoots and total number of leaves of all shoots were estimated 20 dpi and corrected for the same measures at 0 dpi. Total number of flowers of all shoots over the season based on repeated estimates until early autumn (square‐root transformed). Dry weight of all shoots estimated at harvest in the autumn when all leaves had fallen off.

In‐line with the results under controlled conditions, total shoot length and total number of leaves were positively correlated (Pearson *r = *0.439, *df* = 67, *p* < 0.001). Total shoot length also correlated positively with number of flowers (*r = *0.335, *df* = 72, *p* = 0.004). We found no other significant correlations between field estimated traits (*p* > 0.18).

## 4 Discussion

In this study on tolerance to infection by *P. infestans* in the wild potato relative *S. dulcamara*, we detected a nonlinear relationship between shoot growth and degree of infection. Maximum shoot growth occurred at intermediate levels of infection in two out of four investigated susceptible genotypes. This result suggests overcompensation to infection in these genotypes. The relationship between number of leaves produced and degree of infection was also nonlinear, but decreasing. This reduction differed significantly among susceptible genotypes, indicating genetic variation for tolerance to infection. Our field study, involving the overcompensating genotypes, showed increased plant biomass in plants sprayed with *P. infestans*, in‐line with the results from the controlled laboratory condition.

Genetic variation for tolerance to infection has previously been described in several crops (Bingham et al., 2009; Newton, 2016) and wild species (Hochwender, Marquis, & Stowe, 2000; Roux et al., 2010; Simms & Triplett, 1994). We have not, however, found any study that investigated the relationship between disease severity and host fitness or yield in a *Solanum* host or involving *P. infestans*. In our *P. infestans* laboratory infections of *S. dulcamara*, two of the four susceptible genotypes (batch 1) showed a nonlinear relationship between shoot growth and degree of infection, while only genotype differences were significant in the additional two genotypes (batch 2). This may suggest genetic variation in tolerance among genotypes, but because we did not conduct laboratory infections of all genotypes at the same time due to space limitations, we were not able to test for genotype differences among all four susceptible genotypes simultaneously. We judge it highly probable that the differences between batches were caused by genetic differences, as the environmental conditions were controlled and experiments for each batch and genotype were repeated 4–7 times over time. For the second estimated performance trait, number of leaves, we also detected a nonlinear relationship with degree of infection. In this case, the result for the two batches was similar, with clear differences in the relationship for the two genotypes per batch. Thus, this result indicates genetic variance in tolerance.

Overcompensation is a commonly observed response to herbivory (Agrawal, 2000) but has also been shown for infection by virus, bacterial, oomycete and fungal pathogens (both biotrophs and necrotrophs) in *Arabidopsis thaliana* (Hily, Poulicard, Mora, Pagán, & García‐Arenal, 2016; Korves & Bergelson, 2004; Salvaudon, Heraudet, & Shykoff, 2008) and *Brassica* species (Bradley, Gilbert, & Martiny, 2008). For example, in *A. thaliana* infection by the natural biotrophic oomycete pathogen *Hyaloperonospora arabidopsis* resulted in increased seed set in one out of six susceptible inbreed lines (Salvaudon et al., 2008). In the current study on *S. dulcamara*, and the hemibiotrophic oomycete *P. infestans,* shoot length was maximal at intermediate levels of infection in the two susceptible genotypes in batch 1, which is in accordance with overcompensation. Despite a nonlinear relationship with degree of infection also for number of leaves, this performance trait decreased even at low levels of infection. Shedding leaves is a common plant stress response (Chaves, Maroco, & Pereira, 2003). To confirm the laboratory results, we also performed a field study involving the three genotypes of batch 1. Field plants did not show any sign of infection after spraying with inoculum. The lack of disease symptoms may be related to environmental influence on plant resistance, as previously found in field‐grown plants (Abreha et al., 2018), for example, caused by production of smaller and thicker leaves. However, biomass of plant branches harvested at the end of the season increased in sprayed plants compared to unsprayed plants. This result is in‐line with the overcompensation detected in the laboratory. It should be noted that our results are based on infections by one isolate of *P. infestans*. To get large variation in degree of infection, which was crucial for our measure of tolerance, we chose to vary the sporangia concentration rather than using several isolates with different levels of aggressiveness. It is possible that other isolates could have interacted with *S. dulcamara* differently, which would be interesting to evaluate in future studies.

Mechanisms of tolerance are generally poorly understood (Koch et al., 2016; Peterson et al., 2017). It has been suggested that compensation for damage caused by herbivory or pathogen infection could consist of several factors. These include increased chlorophyll concentrations, increased nutrient uptake, increased use of stored resources, delayed flowering and senescence, increased size or number of tissues such as leaves, modified phytohormone balance and altered resource allocation patterns between roots and shoots, or between growth and reproduction (Koch et al., 2016; Mitchell et al., 2016; Pagán & García‐Arenal, 2018; Peterson et al., 2017; Strauss & Agrawal, 1999; Tiffin, 2000). In *S. nigrum*, herbivory was shown to lead to down‐regulation of the hormone system and a subsequent increase in root allocation, which may favor competition with other plants following herbivory (Schmidt & Baldwin, 2009). Even though we did not study tolerance mechanisms in *S. dulcamara*, we hypothesize that the detected increased shoot length in some genotypes is a consequence of longer internodes between leaves, as number of leaves did not increase with shoot length. However, we did not measure internode length. Plants are well‐known to produce longer internodes as a shade avoidance strategy (Pierik & De Wit, 2014). Interestingly, in the perennial sedge *Carex arenaria* it has been shown that rhizomes exposed to soil pathogens can reduce branching and instead grow longer faster to escape infected soil patches (D'Hertefeldt & van der Putten, 1998). In our laboratory experiment, we were only able to estimate relatively early performance traits indicating plant vegetative growth, as plants became too large to keep longer in the infection growth room. Comparing these early traits to later traits in the field suggested that early plant growth was positively correlated with flower production. While it is generally difficult to estimate traits that represent life‐time fitness (Walsh & Blows, 2009), this result suggests that the early performance traits estimated in the laboratory are connected to a component of female fitness. Given that *S. dulcamara* is partially outcrossing (Eijlander & Stiekema, 1994), the number of flowers produced could also be important for pollinator visits and hence male fitness. Moreover, our previous studies in *S. dulcamara* showed a positive correlation between dry weight at the end of the season and number of berries produced over the season (Abreha et al., 2018), indicating that the overcompensatory response detected in the field is connected to female fitness, at least under the given experimental conditions with low levels of infection.

Resistance and tolerance are mutually redundant traits; a fully resistant host cannot increase its fitness by evolving tolerance, and vice versa. Consequently, natural selection should favor high resistance and low tolerance, or low resistance and high tolerance, or a mixed strategy with intermediate values of both traits, but not maximal values of both types of defense (Fornoni, Núñez‐farfán, Valverde, & Rausher, 2004). For example, in *Datura stramonium* exposed to multiple herbivores (a specialist and a generalist), a mixed strategy of reduced resistance and increased tolerance was selected for. This is presumably because resistance was not sufficient for defense against two different herbivores (Carmona & Fornoni, 2013). In our experiment on *S. dulcamara*, we evaluated plant material that ranged from highly resistant to susceptible, in‐line with previous results from detached leaf assays (Abreha et al., 2018). Therefore, in two out of six genotypes we were unable to evaluate tolerance in whole‐plant assays because plants were almost completely resistant. The four susceptible genotypes did not show large differences in resistance, making it hard to explore a potential association between defense strategies. Interestingly, the more resistant genotype included in the field study also increased in weight at the end of the season. We hypothesize that natural selection would favor some susceptibility to *P. infestans *if this leads to fitness benefits. However, it is clear that additional studies are needed to better understand how plant defense strategies to *P. infestans *are linked in *S. dulcamara*.

In conclusion, our laboratory study on wild *S. dulcamara* infected with *P. infestans *showed genetic variation in tolerance. In two genotypes we also found evidence for overcompensation. In the complementary field study, all three investigated genotypes had higher biomass at the end of the season following spraying with the pathogen. This result is in‐line with the overcompensation detected in the laboratory. In future studies, it would be of great interest to explore the potential mechanism and genetics behind our result in *S. dulcamara*. So far, no study has identified genes contributing to variation in tolerance to pathogen infection in any organism (Medzhitov, Schneider, & Soares, 2012; Peterson et al., 2017; Soares, Teixeira, & Moita, 2017). Increased knowledge of the genetics of tolerance to pathogens in wild study systems can provide a better general understanding of the selective forces of resistance versus tolerance and their evolutionary consequences. This knowledge could also provide key information regarding novel and more durable defense strategies. Interestingly, a recent study in potato suggested that overcompensatory growth in response to insect damage on tubers could increase yield for low levels of pest pressure (Poveda, Díaz, & Ramirez, 2018). These types of plant protection strategies are predicted to also be low‐cost strategies and, therefore, have the potential to be implemented in crops exposed to severe diseases caused by rapidly evolving pathogens that are likely to vary with environmental factors, such as *P. infestans*.

## CONFLICT OF INTERESTS

The authors have no competing interests.

## AUTHOR CONTRIBUTIONS

Å.L. and L.R. conceived the ideas; All authors designed methodology; L.M and Å.L. collected the data; Å.L. and L.R. analysed the data and led the writing of the manuscript. All authors contributed critically to the drafts and gave final approval for publication.

## Data Availability

Data available from the Dryad Digital Repository: https://doi.org/10.5061/dryad.8pb0b1f.
